# Basement membrane-related MMP14 predicts poor prognosis and response to immunotherapy in bladder cancer

**DOI:** 10.1186/s12885-024-12489-y

**Published:** 2024-06-19

**Authors:** Xuezhou Zhang, Baoan Hong, Hongwei Li, Jiahui Zhao, Mingchuan Li, Dechao Wei, Yongxing Wang, Ning Zhang

**Affiliations:** 1 Department of Urology, Beijing Anzhen Hospital, Capital Medical University, 2 Anzhen Road, Chaoyang District, Beijing, 100029 P. R. China; 2 Department of Pathology, Key Laboratory of Carcinogenesis and Translational Research (Ministry of Education/Beijing), Peking University Cancer Hospital & Institute, Beijing, P. R. China

**Keywords:** Bladder cancer, Basement membrane, MMP14, Immunohistochemistry, Immunotherapy

## Abstract

**Background:**

Basement membrane (BM) is an important component of the extracellular matrix, which plays an important role in the growth and metastasis of tumor cells. However, few biomarkers based on BM have been developed for prognostic assessment and prediction of immunotherapy in bladder cancer (BLCA).

**Methods:**

In this study, we used the BLCA public database to explore the relationship between BM-related genes (BMRGs) and prognosis. A novel molecular typing of BLCA was performed using consensus clustering. LASSO regression was used to construct a signature based on BMRGs, and its relationship with prognosis was explored using survival analysis. The pivotal BMRGs were further analyzed to assess its clinical characteristics and immune landscape. Finally, immunohistochemistry was used to detect the expression of the hub gene in BLCA patients who underwent surgery or received immune checkpoint inhibitor (ICI) immunotherapy in our hospital.

**Results:**

We comprehensively analyzed the relationship between BMRGs and BLCA, and established a prognostic-related signature which was an independent influence on the prognostic prediction of BLCA. We further screened and validated the pivotal gene-MMP14 in public database. In addition, we found that MMP14 expression in muscle invasive bladder cancer (MIBC) was significantly higher and high MMP14 expression had a poorer response to ICI treatment in our cohort.

**Conclusions:**

Our findings highlighted the satisfactory value of BMRGs and suggested that MMP14 may be a potential biomarker in predicting prognosis and response to immunotherapy in BLCA.

**Supplementary Information:**

The online version contains supplementary material available at 10.1186/s12885-024-12489-y.

## Introduction

Bladder cancer (BLCA) is one of the most common malignant tumors of the urinary system, directly threatening human health and survival [1]. For early-stage BLCA, surgical resection is currently the main treatment modality, but there is a high risk of metastasis and death in advanced BLCA, for which there is a lack of effective means of control [2]. In recent years, immunotherapy based on immune checkpoint inhibitor (ICI) such as programmed cell death ligand 1 (PD-L1)/ programmed cell death protein 1 (PD-1) has become the most promising treatment for advanced BLCA, and has been called a major breakthrough in the past 30 years for BLCA treatment [3, 4]. Unfortunately, most BLCA patients show poor response to immunotherapy and individual PD-L1 levels could not accurately predict the efficacy of immunotherapy [5]. Therefore, it is challenging and urgent to identify novel and reliable markers to predict prognosis and immunotherapy efficacy in BLCA.

The basement membrane (BM), a critical component of the extracellular matrix (ECM), is composed of collagen, laminin, proteoglycan (PG), and fibronectin (FN) [6]. It plays a pivotal role in maintaining tissue structure and integrity. It has been demonstrated that MMPs, as important enzymes known to degrade ECM, play an important role in mediating the processes of tumor angiogenesis, metastasis and invasion. In epithelial cancers, tumor cells often disrupt BM structure by secreting ECM remodeling enzymes such as matrix metalloproteinases (MMPs), facilitating invasion and progression [7]. Recent research has underscored the significance of BM in various solid tumors, including breast cancer, renal cell carcinoma, and lung adenocarcinoma, where alterations in BM composition and integrity correlate with tumor aggressiveness and patient prognosis [8–10]. Despite this, the potential of BMRGs as biomarkers for predicting clinical outcomes in BLCA has not been extensively explored.

Hence, in this study we constructed a BMRGs-based prognostic signature and found that MMP14 was a hub gene in the BMRGs-based signature of BLCA. In addition, we collected two cohorts from Peking University Cancer Hospital & Institute (PUCHI) with patients who received surgery or immunotherapy, demonstrated that MMP14 was closely related to T stage, prognosis and response to ICI immunotherapy in BLCA.

## Materials and methods

### Data collection and preparation

Basement membrane-related genes (BMRGs) were screened and identified from the UniProt database (accessed on 12 June 2023). RNA-Seq expression data and clinical details for BLCA patients were obtained from two sources: the TCGA-BLCA cohort within The Cancer Genome Atlas database (accessed on 15 June 2023) and the GEO BLCA cohort (GSE13507). The clinical data encompassed age, gender, stage, TNM stage, survival status, and overall survival (OS). Inclusion criteria stipulated that patients had a histopathological diagnosis of BLCA, complete survival records, and comprehensive clinical information.

Additionally, the IMvigor210 study, a phase II clinical trial evaluating PD-L1 ICI (Atezolizumab) in locally advanced or metastatic urothelial cancer after platinum-based chemotherapy failure, was incorporated [11]. In total, this research encompassed 401 BLCA samples from TCGA, 169 samples from GSE13507, and 195 samples from IMvigor210. Data on immune cell fractions were retrieved from the Tumor Immune Estimation Resource (TIMER) website (accessed on 21 June 2023) [12].

### Identification of prognosis-related BMRGs

In the TCGA-BLCA cohort, differentially expressed BMRGs between normal and BLCA samples were identified using the R package “limma”. The filtering criteria included a false discovery rate (FDR) < 0.05 and an |log2FC| > 1. The most significantly up- and downregulated genes were visualized using a volcano plot and heatmap.

The research team further examined the association between BMRGs’ expression levels and BLCA patient survival in both the TCGA-BLCA and GSE13507 cohorts. Additionally, a network diagram was created to illustrate the relationship between the expression levels of the top prognostic BMRGs. Using the R package “survival”, univariate Cox regression analysis was performed to pinpoint prognostic BMRGs.

The Genomic Data Commons (GDC) TCGA BLCA copy number dataset was obtained from the UCSC XENA database (accessed on 21 June 2023), and the R package “RCircos” was utilized to assess the frequency of copy number variations (CNV) and chromosomal alterations in prognosis-related BMRGs.

### Identification of the BM-related patterns by consensus clustering

Building on the identified BMRGs, we employed a consensus clustering approach to uncover novel BM-related patterns within the TCGA-BLCA cohort and stratify patients. Principal component analysis (PCA) was used to extract data from the consensus matrix, aiding in the determination of the optimal number of clusters. The empirical cumulative distribution function (ecdf) method was applied to generate a fitting curve, and the minimum area under the cumulative distribution function (CDF) curve was computed to select the optimal K value for clustering. BLCA patients were then grouped accordingly. Kaplan-Meier analysis was conducted to assess survival differences between the clusters. The accuracy of these clusters was further validated using PCA, UMAP, and tSNE analyses.

### Clinical characterization and immunological landscapes of clusters

The expression heatmaps and corresponding clinical pathological features of BMRGs related to prognosis were analyzed, and the expression patterns of these BMRGs in different groups were displayed using box plots. We analyzed the expression of BMRGs in different clusters and also showed the pattern of immune infiltration in different subtype clusters. Gene Set Variation Analysis (GSVA) and Gene Set Enrichment Analysis (GSEA) were conducted using R software packages “GSVA” and “GSEA Base”, with a focus on analyzing the differences in KEGG pathway enrichment between the aforementioned clusters.

### Construction of a BMRGs-based signature and nomogram

The research team employed univariate Cox regression analysis using the R package “survival” to pinpoint BMRGs linked to prognosis. Subsequently, these genes were integrated into the Lasso Cox model for cross-validation using the “glmnet” package in R, culminating in the creation of a prognostic signature derived from BMRGs to predict the outcome for BLCA patients. The risk score, formulated as the summation of (βi×Expi) for each BMRG, was utilized. Heatmaps effectively illustrated the correlation between risk scores and signature genes.

To quantitatively assess the BM-related patterns in BLCA patients, a scoring system was devised for BMRGs. Patients were stratified into high- and low-risk groups based on the median risk score. Kaplan-Meier analysis was employed to compare the overall survival (OS) between these groups. The prognostic accuracy of the signature was gauged by the area under the curve (AUC) values. Furthermore, patients with comprehensive clinical data were selected to ascertain the signature’s independence in prognostic prediction. The prognostic significance of the BMRG-based signature was evaluated using multivariate Cox regression analysis.

Clinicopathological characteristics and risk score of BLCA patients were used to construct the nomogram. The Time-C index was used to validate the predictive performance of the nomogram. In addition, the researchers plotted calibration curves to assess the agreement between predicted and actual survival and performed decision curve analysis (DCA) to assess the net clinical benefit of BLCA patients [13].

### Identification and validation of the hub gene of BMRGs

Differentially expressed BMRGs were further put into the Search Tool for the Retrieval of Interacting Gene (STRING, https://string-db.org/, accessed date on 23 June 2023) to construct protein-protein interactions (PPI) network. Cytoscape was used to screen the PPI network and identify the hub gene in the PPI network.

The top 20 genes in the differentially expressed BMRGs were screened as candidates by using the |logFC| value as a reference. The BMRGs in the PPI network were sorted using the Maximal Clique Centrality (MCC) method of the cytoHubba plugin [14], and the genes with higher scores were also selected as candidates. Then we used the Venn diagram to take the intersection of the above two candidate gene sets, and the gene with the highest MCC score among the intersected genes was recognized as the hub gene.

### Clinicopathologic features and immunoscape of the hub gene

The TCGA-BLCA cohort was further used to validated the hub gene’s expression in BLCA and normal samples by using Gene Set Cancer Analysis (GSCA) online website (http://bioinfo.life.hust.edu.cn/GSCA/#/, accessed date on 26 June 2023) and R software [15]. Correlations between hub gene and prognosis, clinicopathologic features (age, gender, grade, TNM stage, stage), and pathway activity of TCGA-BLCA patients were analyzed using R software.

TIMER database could be used for tumor-infiltrating immune cell analysis. The immune cell fraction data was downloaded from the TIMER online website (http://cistrome.dfci.harvard.edu/TIMER/, accessed date on 21 June 2023) [12]. Then, the researchers analyzed the hub gene and its relationship with the response of immunotherapy in IMvigor210-BLCA cohort and The Cancer Immunome Atlas (TCIA) online website (https://tcia.at/home, accessed date on 27 June 2023) [16].

### Clinical validation by immunohistochemistry (IHC)

In this investigation, we enrolled 68 BLCA patients who underwent either transurethral resection of bladder tumor (TURBT) or radical cystectomy (RC) at Peking University Cancer Hospital & Institute (PUCHI) between October 2012 and December 2021. Strict inclusion criteria were followed: BLCA confirmation by pathology, no prior adjuvant therapy before surgery, and complete clinical and pathological records for patients aged over 18. Informed consent was obtained, and the study adhered to ethical guidelines approved by the ethics committee of Peking University Cancer Hospital (Institutional Review Board approval number: 2020KT143-GZ01). All clinical characteristics are summarized in Table 1.

**Table 1:** Correlation between clinicopathological features and MMP14 expression in PUCHI-BLCA cohort

Clinical features		Total:68(%)	MMP14 expression		*P*-value*
			negative	positive	
Age	< 60	19(27.94%)	4	15	
	≥ 60	49(72.06%)	20	29	0.1629
Gender	Male	43(63.24%)	13	30	
	Female	25(36.76%)	11	14	0.2983
T	T1	22(32.35%)	15	7	
	T2-T4	46(67.65%)	9	37	0.0003
N	N0	48(70.59%)	20	28	
	N1-N3	20(29.41%)	4	16	0.1035
M	M0	59(86.76%)	23	36	
	M1	9(13.24%)	1	8	0.1442

*Fisher’s exact test

Additionally, 46 BLCA patients receiving ICI immunotherapy, either as monotherapy or combined with platinum-based chemotherapy at PUCHI between November 2019 and September 2021, were included. Treatment response was assessed using the immune-modified Response Evaluation Criteria in Solid Tumors (iRECIST) v1.1, including complete response (CR), partial response (PR), stable disease (SD), and disease progression (PD). Table 2 summarizes the clinical information and efficacy assessments. Immunohistochemical staining was performed on paraffin-embedded tissue sections using an MMP14 polyclonal antibody (Proteintech company, ID: No. 14552-1-AP). Two senior pathologists independently scored and analyzed the samples.

**Table 2:** Clinical information, efficacy evaluation and MMP14 expression of the 46 BLCA patients treated with ICI in PUCHI cohort

Clinical features		Total:46(%)	MMP14 expression		*P*-value*
			low	high	
Age	< 60	18(39.13%)	6	12	
	≥ 60	28(60.87%)	12	16	-
Gender	Male	24(52.17%)	8	16	
	Female	22(47.83%)	10	12	-
T	T1	7(15.22%)	6	1	
	T2	10(21.74%)	3	7	
	T3	22(47.83%)	7	15	
	T4	5(10.87%)	1	4	
	Tx	2(4.35%)	1	1	-
N	N0	8(17.39%)	4	4	
	N1	13(28.26%)	4	9	
	N2	15(32.61%)	6	9	
	N3	8(17.39%)	3	5	
	Nx	2(4.35%)	1	1	-
M	M0	17(36.96%)	7	10	
	M1	25(54.35%)	9	16	
	Mx	4(8.70%)	2	2	-
Efficacy	CR	2(4.35%)	1	1	
	PR	8(17.39%)	6	2	
	SD	19(41.30%)	6	13	
	PD	17(36.96%)	5	12	0.0332(CR + PR vs. SD + PD)

*Fisher’s exact test

### Statistical analysis

Statistical analyses were performed using R software (version 4.0.2), with *p* < 0.05 denoting significance. To ensure the integrity and dependability of our statistical findings, we applied multiple test corrections for differential expression analysis and batch corrections across different datasets.

## Results

### Identification of BMRGs related to prognosis in BLCA

**Fig. 1:**
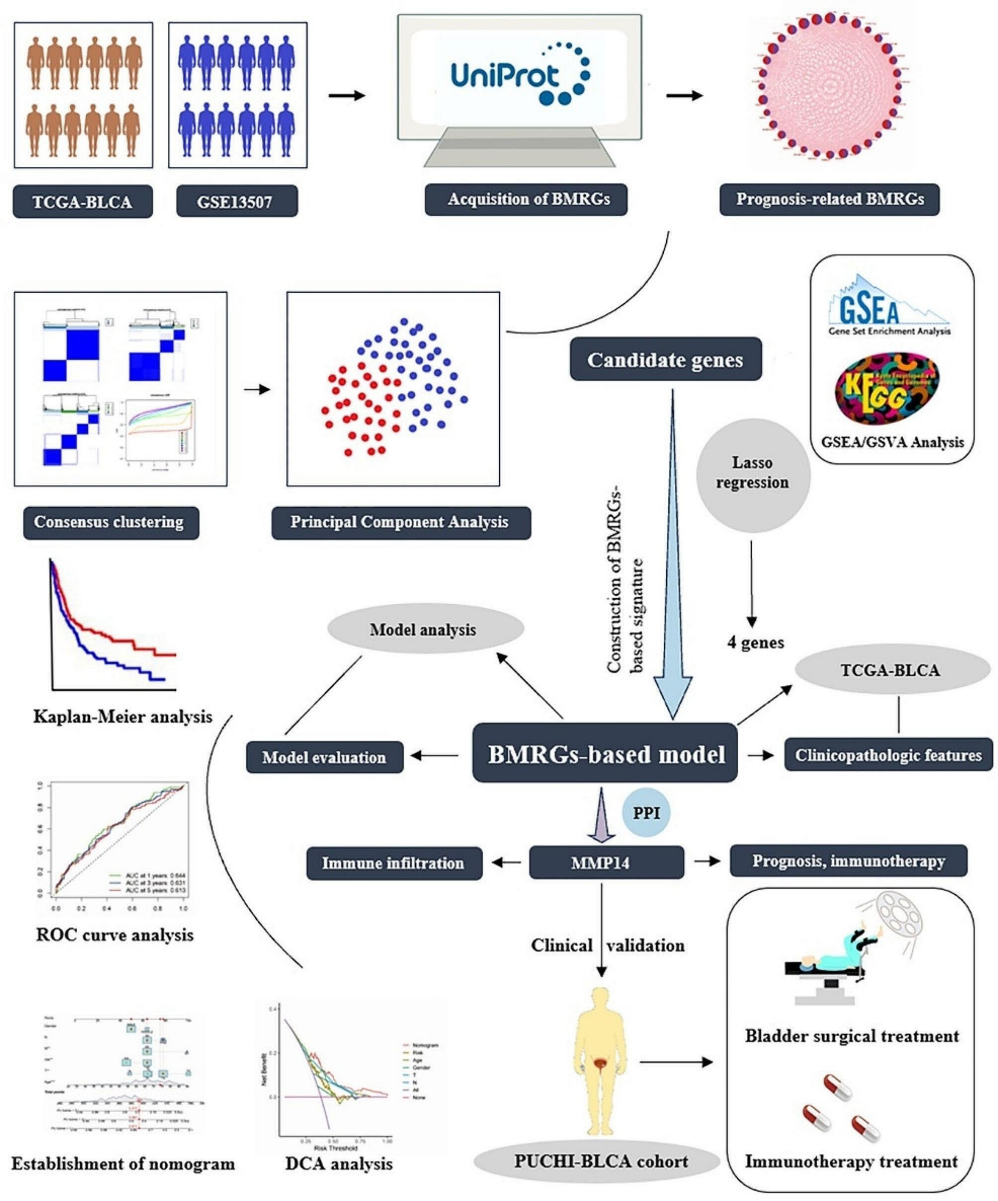
Shows the flowchart of our study

**Fig. 2:**
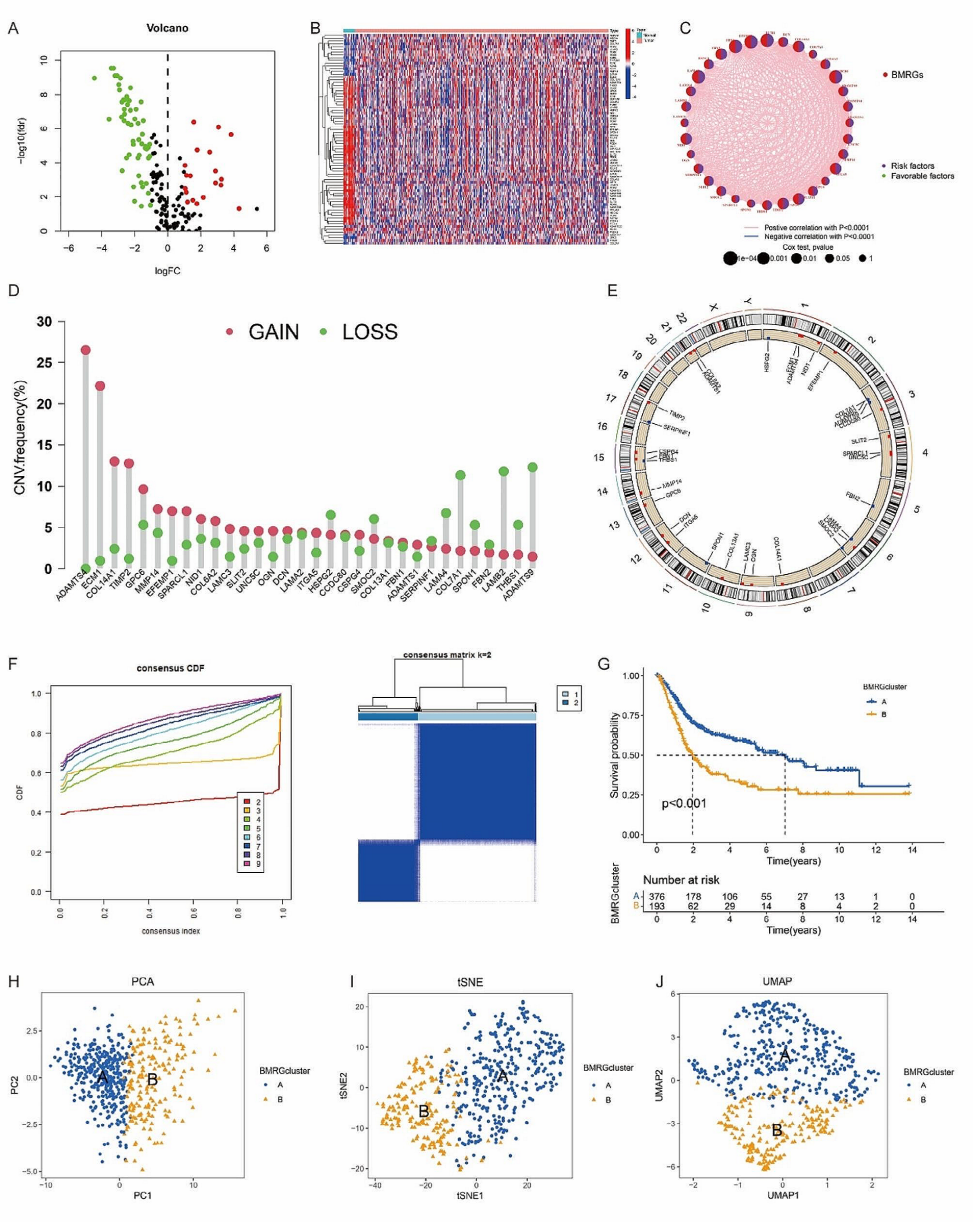
(**A**) All the differential BMRGs was displayed in the volcano map; (**B**) The most significant up-regulated and down-regulated genes; (**C**) The network plot showed the relationship between the expression levels of the top prognosis-related BMRGs of rank; (**D, E**) The copy number variation frequency (CNV.frequency%) and the chromosome region and alteration of the above prognosis-related BMRGs; (**F**) Consensus clustering of prognosis-related BMRGs, when k = 2, the cohort could be well classified into two subtypes; (**G**) Overall survival analysis showed a significant difference in prognosis between the two subtypes (*p* < 0.001) and cluster B has a worse overall survival; (**H-J**) PCA, tSNE and UMAP analyses were used to test the accuracy of this clustering

Figure 1 outlines the flowchart of our study. A total of 222 BMRGs were sourced from the UniProt database (supplementary Table 1). Differential expression analysis between BLCA and normal samples, using the R package “limma”, yielded 76 BMRGs (supplementary Table 2). The volcano plot in Fig. 2A showcases all differential genes, highlighting the most prominent up- and downregulated genes in Fig. 2B. Prognostic analysis of these genes revealed 32 significant prognostic BMRGs (*p* < 0.05) (supplementary Table 3), with their expression relationships visualized in the network plot (Fig. 2C). Additionally, Fig. 2D **and** Fig. 2E illustrate the copy number variation frequency and chromosomal alterations of these prognostic BMRGs.

### Consistent clustering of BLCA molecular subgroups using BMRGs

To further elucidate the role of BMRGs in BLCA, consensus clustering was performed using the R package “Consensus Cluster Plus.” As depicted in Fig. 2F, optimal classification occurred at k = 2, resulting in two distinct subtypes with significantly different prognoses (*p* < 0.001), where cluster B exhibited poorer overall survival (Fig. 2G). PCA, tSNE, and UMAP analyses confirmed the accuracy of this clustering (Fig. 2H-J).

**Fig. 3:**
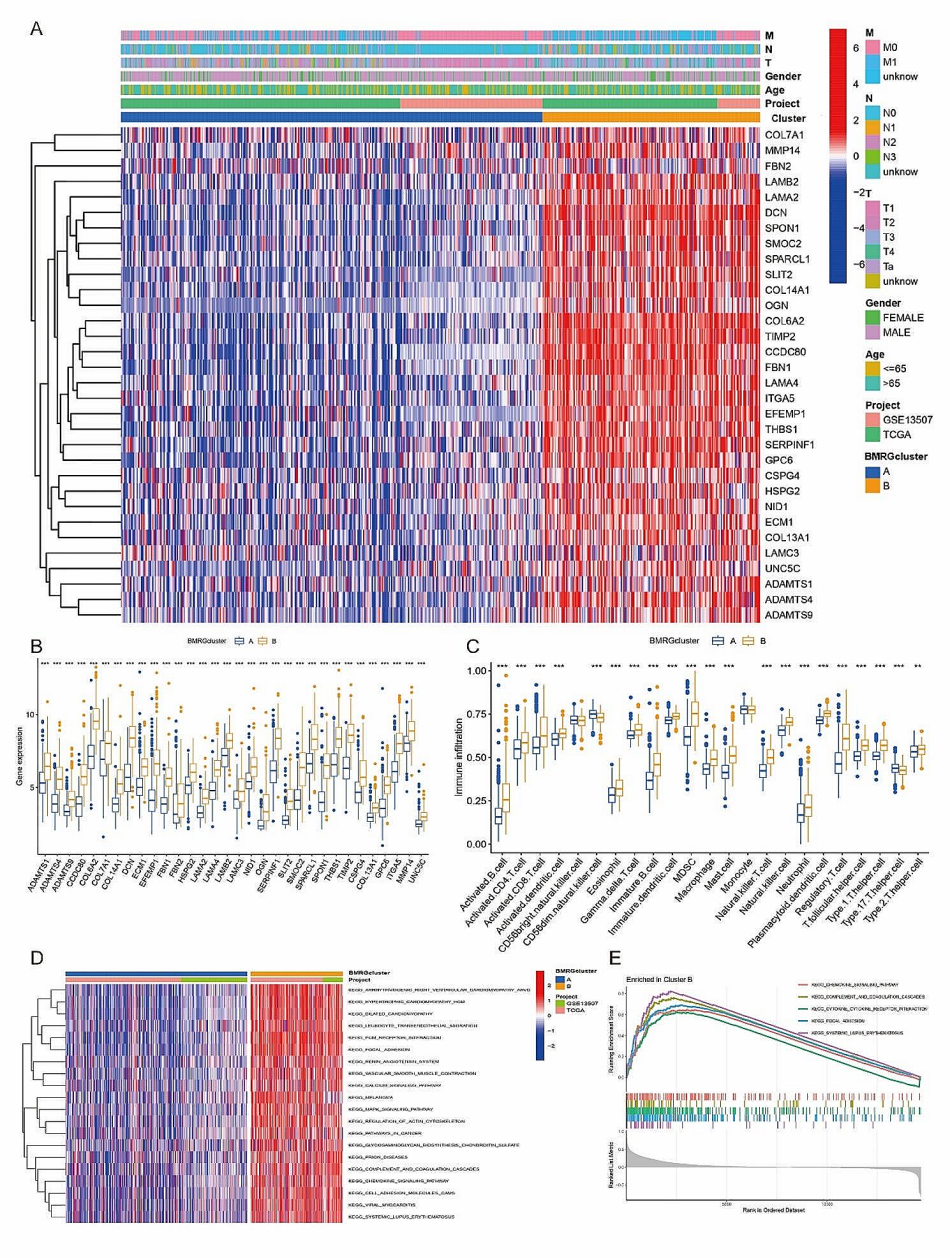
(**A**) Heat map was showed of the above BMRGs expression and corresponding clinicopathological features of two subtypes; (**B**) Boxplot was also used to show the expression patterns of BMRGs in the two subtypes (∗*p* < 0.05; ∗∗*p* < 0.01; ∗∗∗*p* < 0.001); (**C**) Immune infiltration patterns in the two subtype clusters (∗*p* < 0.05; ∗∗*p* < 0.01; ∗∗∗*p* < 0.001); (**D**) The differential enrichment of KEGG pathways between clusters A and B; (**E**) GSEA enrichment analysis and the top 5 most significant pathways with Cluster B

Figure 3A presents a heatmap of BMRG expression and corresponding clinicopathological features across the two subtypes, while Fig. 3B visualizes expression patterns of BMRGs within these subtypes. Immune infiltration patterns are identified in Fig. 3C. The pathway enrichment results demonstrated key differences in KEGG pathways between clusters A and B (Fig. 3D). Cluster B, associated with poorer prognosis, was predominantly involved in “ECM receptor interaction” and “focal adhesion” pathways, crucial for tumor invasion and metastasis. GSEA enrichment analysis further emphasized these findings, with the top 5 significant pathways in Cluster B displayed in Fig. 3E.

### Construction and validation of BMRGs-based prognostic model

**Fig. 4:**
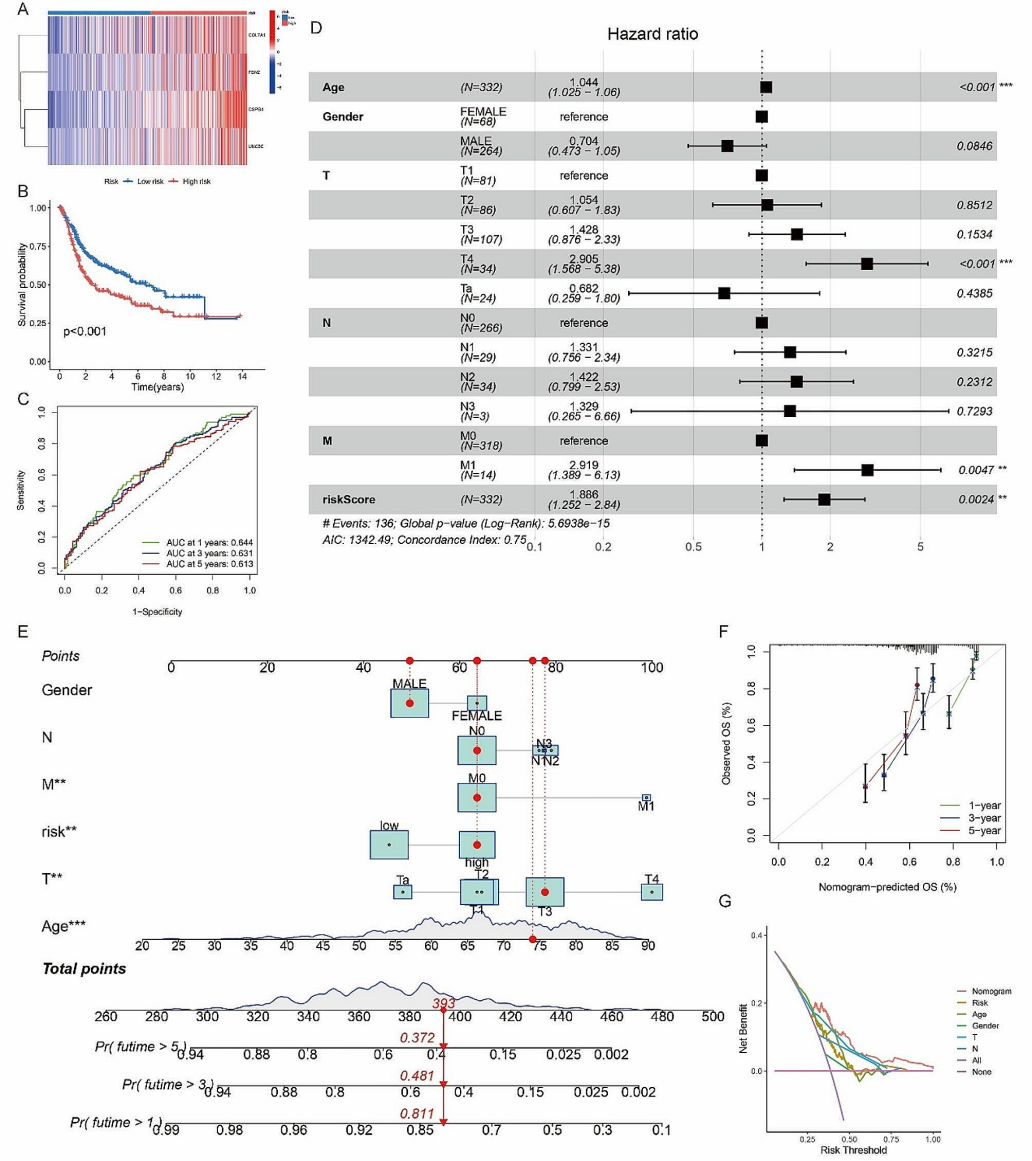
(**A**) The heat map indicated the expression patterns of the 4 hub BMRGs; (**B**) K-M survival curve showed that the BMRGmodel was closely related to the OS of BLCA patients (*p* < 0.001); (**C**) ROC curve analysis for OS at 1-, 3-, and 5- years of the BMRGmodel; (**D**) Multivariate Cox regression analysis confirmed that the BMRGmodel was an independent prognostic factor for BLCA (*p* = 0.0024) (∗*p* < 0.05; ∗∗*p* < 0.01; ∗∗∗*p* < 0.001); (**E**) Establishment of a prognostic nomogram for BLCA patients (∗*p* < 0.05; ∗∗*p* < 0.01; ∗∗∗*p* < 0.001); (**F**) The calibration plot for predicting 1-, 3-, and 5- years survival; (**G**) Decision curve analysis (DCA) for predicting survival in BLCA patients

A prognostic model, BMRGmodel, was constructed using Lasso regression analysis, encompassing 4 BMRGs (COL7A1, FBN2, CSPG4, and UNC5C). The heatmap in Fig. 4A illustrates the expression patterns of these hub BMRGs. The risk score based on BMRGs = COL7A1’s expression level × 0.0805978623968319 + FBN2’s expression level × 0.113399875769463 + CSPG4’s expression level × 0.138933668778148 + UNC5C’s expression level × 0.191098689380473. Risk scoring based on BMRG expression levels was calculated, and the K-M survival curve demonstrated a strong correlation between the BMRGmodel and OS in BLCA patients (*p* < 0.001) (Fig. 4B). High-risk patients had significantly shorter 5-year survival rates. ROC curve analysis at 1-, 3-, and 5-years confirmed the model’s predictive performance (Fig. 4C), and multivariate Cox regression analysis established the BMRGmodel as an independent prognostic factor for BLCA (*p* = 0.0024) (Fig. 4D).

### Establishment of a prognostic nomogram for BLCA patients

Integration of the BMRGmodel with clinicopathological information resulted in the development of a nomogram (Fig. 4E), which exhibited good predictive performance for 1-, 3-, and 5-year survival (Fig. 4F). Decision curve analysis (DCA) further validated the nomogram’s utility in predicting survival in BLCA patients (Fig. 4G).

### Identification of the hub and its clinicopathologic features

**Fig. 5:**
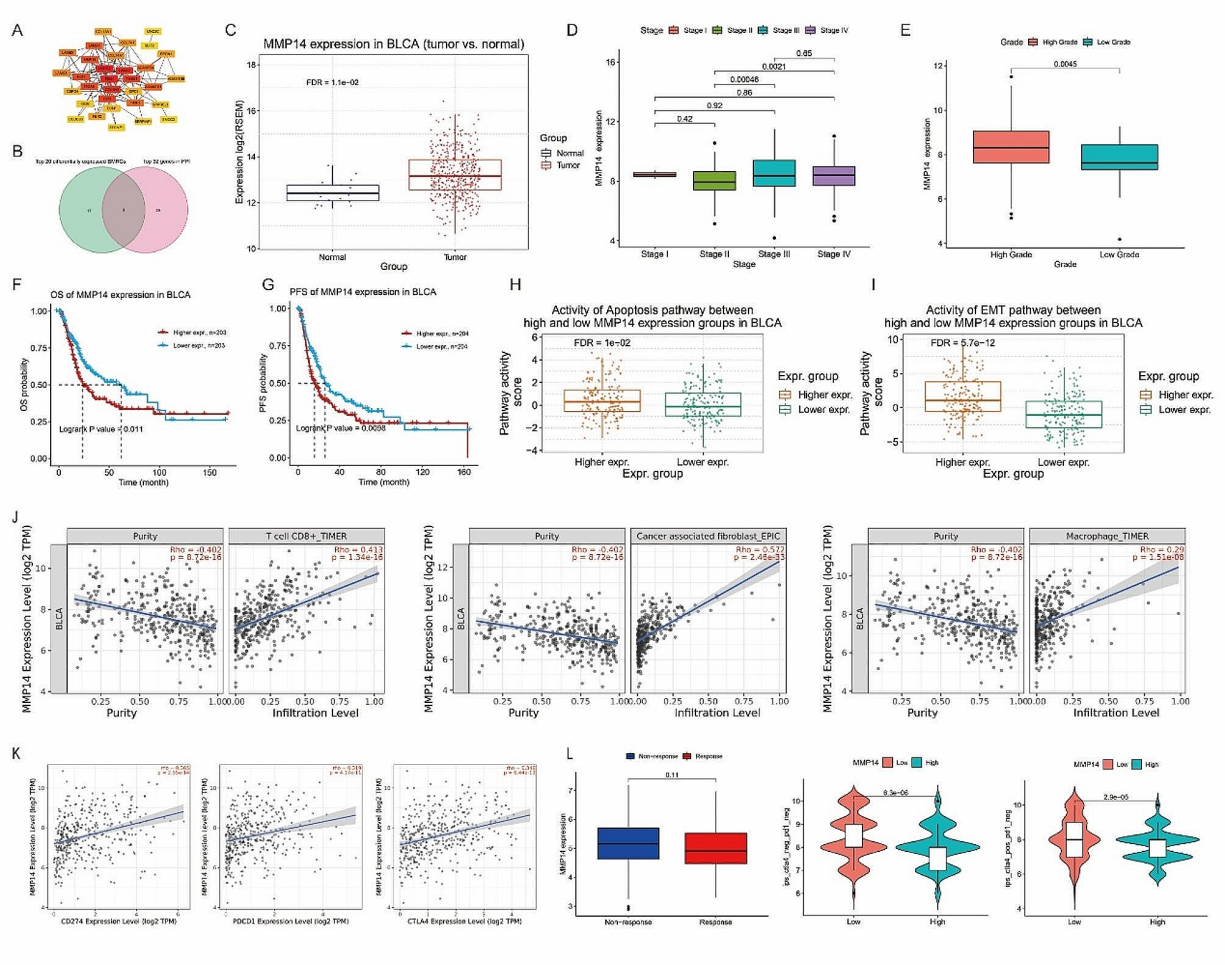
(**A**) The Protein-Protein Interaction (PPI) network and MCC method of the cytoHubba plugin was used to screen and identify the hub gene; (**B**) Venn diagram got three intersecting genes; (**C**) MMP14 was highly expressed in BLCA tissue compared with normal bladder tissue (*p* = 1.32e-3); (**D**) MMP14 expression levels were overall higher in stage II and III; (**E**) MMP14 expression levels in high grade BLCA were more highly than low grade BLCA (*p* = 0.0045); (**F, G**) High expressions of MMP14 had a significantly worse overall survival (OS) rate (*p* = 0.011) and progression free survival (PFS) rate (*p* = 0.0098) compared to patients with low expressions of MMP14; (**H, I**) MMP14 was closely associated with the apoptosis and epithelial-mesenchymal transition (EMT) pathways; (**J**) MMP14 expression level was positively correlated with infiltration level of CD8 + T cells (*R* = 0.413, *p* = 1.34e-16), cancer associated fibroblast (*R* = 0.572, *p* = 2.46e-33) and macrophage (*R* = 0.29, *p* = 1.51e-08); (**K**) MMP14 expression level was positively correlated with CD274 (*R* = 0.365, *p* = 2.55e-14), PDCD1 (*R* = 0.319, *p* = 4.17e-11) and CTLA4 (*R* = 0.346, *p* = 6.44e-13); (**L**) Low MMP14 expression group may have a better response to ICI immunotherapy

To explore interactions among differentially expressed prognosis-related BMRGs in BLCA, hub genes were identified using the MCC method of the cytoHubba plugin (Fig. 5A). A candidate gene set was created by selecting the top 20 differentially expressed BMRGs based on |logFC| values and genes with high MCC scores in the PPI network (supplementary Table 4). Intersection of these sets yielded three genes (MMP14, FBN2, and COL7A1), with MMP14 recognized as the hub gene due to its highest MCC score (Fig. 5B).

Validation analysis revealed significant overexpression of MMP14 in BLCA tissues compared to normal tissues (*p* = 1.32e-3) (Fig. 5C). Higher MMP14 expression levels were observed in stages II and III (Fig. 5D) and high-grade BLCA (*p* = 0.0045) (Fig. 5E). Patients with high MMP14 expression had poorer OS (*p* = 0.011) and progression free survival PFS (*p* = 0.0098) rates (Fig. 5F, G). Pathway analysis implicated that higher MMP14 expression was closely associated with the apoptosis and epithelial-mesenchymal transition (EMT) pathways (Fig. 5H, I).

### Immunoscape and immunotherapy analysis of MMP14

Our further analysis of immune cell content revealed a positive correlation between MMP14 expression and the infiltration levels of CD8 + T cells, cancer-associated fibroblasts, and macrophages (Fig. 5J). This correlation provides valuable insights into the immune microenvironment of BLCA patients. Additionally, we observed a positive correlation between MMP14 expression and immune checkpoint-related genes, including CD274, PDCD1, and CTLA4 (Fig. 5K).

In the IMvigor210 BLCA cohort, although the response to immunotherapy was better in the low MMP14 expression group compared to the high expression group, this difference did not reach statistical significance (*p* = 0.11) (Fig. 5L). However, further evaluation of the relationship between MMP14 expression and the clinical response to PD-1 and CTLA-4 blockers using the immunophenoscore (IPS) indicated a significant correlation between low MMP14 expression and a better response to ICI immunotherapy (Fig. 5L).

### Clinical validation

**Fig. 6:**
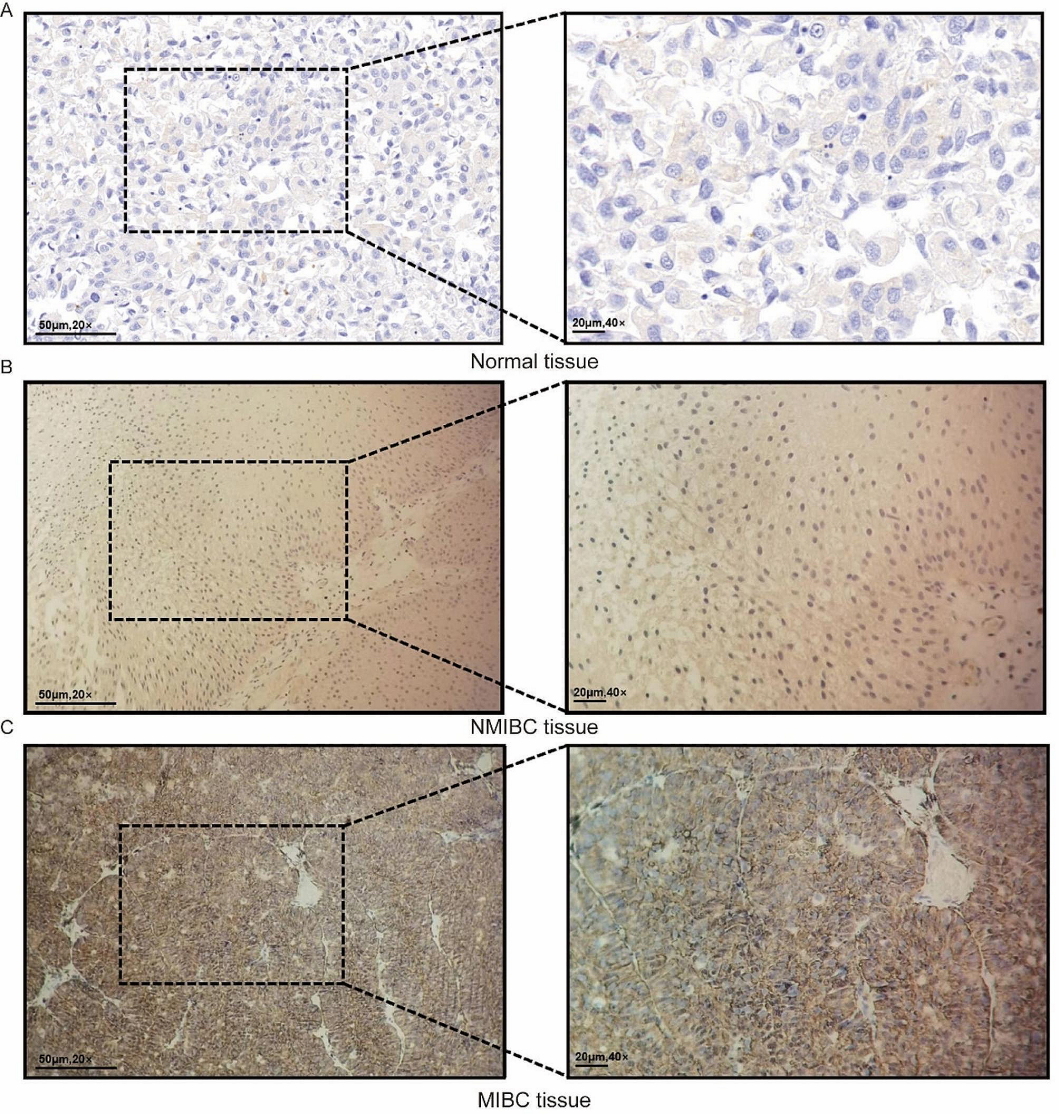
Clinical validation by IHC: (**A**) Normal tissue; (**B**) NMIBC tissue; (**C**) MIBC tissue

To validate our findings, we conducted IHC analysis of MMP14 protein expression in 68 PUCHI-BLCA patients. We found that MMP14 was highly expressed in 64.71% of BLCA tissues (Fig. 6A). Additionally, we observed a significant increase in MMP14 expression in muscle-invasive bladder cancer (MIBC) compared to non-muscle-invasive bladder cancer (NMIBC) (Fig. 6B, C).

To assess the predictive value of MMP14 for clinical response to ICI immunotherapy, we examined MMP14 expression in 46 BLCA patients who received ICI treatment. The results showed a statistically significant difference between the “CR + PR” group and the “SD + PD” group (*p* = 0.0332), indicating that patients with high MMP14 expression had a poorer response to ICI treatment (Table 2).

## Discussion

The incidence of BLCA is high, and muscle invasive bladder cancer (MIBC) currently has limited means of control. In recent years, immunotherapy represented by PD-L1/PD-1 is a hot spot in the field of MIBC treatment, which is now widely used in the clinical treatment of BLCA and has achieved impressive results [17]. However, only detecting the PD-L1 expression level could not meet the clinical needs at present, and the immunotherapy of MIBC lacks markers with high specificity [18]. Therefore, it is urgent and significant to explore novel markers to meet clinical requirements. In this study, we demonstrated for the first time that MMP14 is closely associated with the prognosis and immunotherapy efficacy of BLCA patients based on samples from public databases and our cohort.

The basement membrane (BM) is a specialized extracellular matrix (ECM) component, which is a dense, thin, layer-like structure composed mainly of cell-secreted proteins and polysaccharides, among other components [19, 20]. BM maintains normal tissue morphology and also regulates a variety of cellular behaviors, including cell proliferation, migration, differentiation, and invasion [21]. Studies have shown that abnormalities in BM structure are necessary for tumor cells to metastasize [22]. Functional changes in the BM are always present as tumor cells shed their primary foci, enter the blood circulation system to form circulating tumor cell (CTC) and form distant metastatic foci again [21]. BM was strongly associated with breast cancer, renal cell carcinoma, lung adenocarcinoma, et al. [8–10], while its role in BLCA has not been evaluated. In this study, we demonstrated that BMRGs are closely associated with BLCA patients, which may provide a new direction for the exploration of BLCA.

In order to fully elaborate the role of BMRGs in BLCA, we identified a new molecular typing system based on BMRGs. We identified 2 BMRG-clusters and found that BMRG-cluster B has a worse overall survival and mainly involved in “ECM receptor interaction” and “focal adhesion” pathways, which are crucial pathways for tumor cell invasion and metastasis [23, 24]. GSEA enrichment analysis showed that cluster B was mainly activated in “cytokine-cytokine receptor interaction”, “chemokine signaling pathway”, and “focal adhesion” pathways, confirming the tumor associated-activated inflammatory status in cluster B.

Considering the individual heterogeneity we then established a BMRGs-based model to assess the BM modification pattern of various BLCA patients. The BMRGs-based model was constructed based on four filtered genes: COL7A1, FBN2, CSPG4 and UNC5C. The results showed that the model risk score was significantly related to prognosis, indicated that the imbalanced expression of BMRGs may play different biological roles in the tumorigenesis, progression and tumor microenvironment in BLCA. Studies have shown that high COL7A1 expression is associated with poor prognosis in clear cell renal cell carcinoma (ccRCC), and in vitro knockdown of COL7A1 expression significantly affects the migratory ability of ccRCC cells [25]. Other studies have also shown that FBN2, CSPG4 and UNC5C as oncogenes were significantly associated with poor prognosis, which is in line with our findings [26–28].

To explore the interaction of the prognosis-related BMRGs in BLCA, STRING database was used to construct the PPI network and MMP14 with the highest MCC score among the intersected genes was recognized as the hub gene. MMP14, also known as matrix metallopeptidase 14, is a member of the matrix metalloproteinases (MMPs) family, and its encoded protein is a member of the membrane-type MMP (MT-MMP) subfamily [29]. MMP14 is involved in extracellular matrix catabolism during normal physiological processes such as embryonic development, reproduction, and tissue remodeling, as well as during disease processes such as arthritis and metastasis of tumors [30]. Previous studies have shown that MMP14 plays a key role in the progression of a variety of malignant tumors, including pancreatic cancer, colorectal cancer and intrahepatic cholangiocarcinoma [31–33]. Similarly, MMP14 plays an important role in lung cancer bone metastasis [34]. Our study showed that MMP14 was highly expressed in BLCA and was closely associated with stage, grade and prognosis. Clinical samples in our hospital showed the same results, which provides a new method for the determination of BLCA diagnostic markers and screening of therapeutic targets.

Over the past decades, the accumulated interest in immunotherapy, coupled with a growing understanding the exploration of the pathogenesis in BLCA, has dramatically enriched the therapeutic treatment against advanced BLCA. Interestingly, we found a positive correlation between MMP14 and both CD8 + T cells as well as immune checkpoints in this study. Simultaneous findings based on the IMvigor210 cohort and the TCIA database suggest that MMP14 could predict the response to immunotherapy in BLCA. Further validation using the PUCHI cohort revealed that patients with high expression of MMP14 had a poorer response to immunotherapy, which may provide novel markers and research directions for immunotherapy of BLCA. However, further studies are still warranted to illuminate the specific role and mechanisms of MMP14 in BLCA microenvironment.

In light of the significant role of the BM in tumor progression, our study introduces MMP14 as a pivotal hub gene within the BMRGs cluster impacting BLCA prognosis and response to immunotherapy. Unlike previous studies that focused broadly on MMPs, our study focused on MMP14 and explored its relevance to tumor prognosis and immunotherapy response. The identification of MMP14 not only enriches the biomarker repertoire for BLCA but also opens new avenues for targeted therapies that could disrupt its pathways to improve patient outcomes. Our findings suggest that MMP14 expression correlates with immune cell infiltration and immune checkpoint expression, highlighting its dual role in tumor biology and immune landscape modulation. This dual functionality makes MMP14 a promising target for combinatorial therapy strategies, which predicts the efficacy of immunotherapy and is also strongly associated with BLCA prognosis.

The current research should take into account several limitations. First, as a retrospective study, the number of patients included in our cohort was insufficient, especially the small number of patients who received immunotherapy, which may be statistically different and subject to selection bias. On the other hand, our study focused on the expression and prognostic value of MMP14 in BLCA patients, and bioinformatics studies based on public databases also require further experiments to validate the mechanism of MMP14 involvement in the metastasis, progression, and ICI therapeutic response of BLCA.

## Conclusion

Our findings highlighted the satisfactory value of BMRGs and suggested that MMP14 may be a potential biomarker in predicting prognosis and response of immunotherapy in BLCA.

### Electronic supplementary material

Below is the link to the electronic supplementary material.

Supplementary Material 1

Supplementary Material 2

Supplementary Material 3

Supplementary Material 4

## Data Availability

The dataset supporting the conclusions of this article is available in The Cancer Genome Atlas (TCGA) database (https://tcga-data.nci.nih.gov/tcga/). The total data of GSE13507 dataset acquired from GEO (https://www.ncbi.nlm.nih.gov/geo/) database, IMvigor210 cohort data can be found at http://research-pub.gene.com/IMvigor210CoreBiologies. The data used and analyzed during the current study are available from the corresponding author upon reasonable request.
