# Supplementary material for: Basement membrane-related MMP14 predicts poor prognosis and response to immunotherapy in bladder cancer
Source: BMC Cancer. 2024 Jun 19;24:746. doi: 10.1186/s12885-024-12489-y (PMC11186261; doi:10.1186/s12885-024-12489-y)
Supplement: Supplementary file 1 — Supplementary Material 1 [file 12885_2024_12489_MOESM1_ESM.docx]

**Supplementary Table 1. A total of 222 BMRGs were obtained from the UniProt database.**

| ACAN | COL8A1 | LAD1 | PAPLN | ADAM9 | ITGB7 |
| --- | --- | --- | --- | --- | --- |
| ACHE | COL8A2 | LAMA1 | PODN | ADAM10 | ITGB8 |
| ADAMTS1 | COL9A1 | LAMA2 | POSTN | ADAM17 | MEGF9 |
| ADAMTS2 | COL9A2 | LAMA3 | PTN | CD44 | MMP14 |
| ADAMTS3 | COL9A3 | LAMA4 | PXDN | CD151 | MPZL2 |
| ADAMTS4 | COL12A1 | LAMA5 | PXDNL | CSPG4 | MUSK |
| ADAMTS5 | COL14A1 | LAMB1 | RECK | COL13A1 | PTPRF |
| ADAMTS6 | COL15A1 | LAMB2 | SERPINF1 | DAG1 | RPSA |
| ADAMTS7 | COL17A1 | LAMB3 | SLIT1 | DCC | ROBO1 |
| ADAMTS8 | COL18A1 | LAMB4 | SLIT2 | DDR1 | ROBO2 |
| ADAMTS9 | COL28A1 | LAMC1 | SLIT3 | DDR2 | ROBO3 |
| ADAMTS10 | COLQ | LAMC2 | SEMA3B | EVA1A | ROBO4 |
| ADAMTS13 | CST3 | LAMC3 | SMC3 | EVA1B | SDC1 |
| ADAMTS14 | CTSA | LOXL1 | SMOC1 | EVA1C | SDC4 |
| ADAMTS15 | CTSB | LOXL2 | SMOC2 | GPC1 | TENM1 |
| ADAMTS16 | CTSD | LOXL4 | SPARC | GPC2 | TENM2 |
| ADAMTS17 | DCN | LUM | SPARCL1 | GPC3 | TENM3 |
| ADAMTS18 | ECM1 | MATN1 | SPOCK1 | GPC4 | TENM4 |
| ADAMTS19 | EFEMP1 | MATN2 | SPOCK2 | GPC5 | UNC5A |
| ADAMTS20 | EFEMP2 | MATN4 | SPOCK3 | GPC6 | UNC5B |
| AGRN | EGFL6 | MEP1A | SPON1 | ITGA1 | UNC5C |
| AMELX | EGFLAM | MEP1B | SPON2 | ITGA2 | UNC5D |
| AMTN | FBLN1 | MMP1 | TGFB2 | ITGA2B |  |
| ANG | FBLN2 | MMP2 | TGFB1 | ITGA3 |  |
| BCAN | FBLN5 | MMP7 | TGFBI | ITGA4 |  |
| BGN | FBN1 | MMP17 | THBS1 | ITGA5 |  |
| CCDC80 | FBN2 | MMP21 | THBS2 | ITGA6 |  |
| CERT1 | FBN3 | MMP26 | THBS4 | ITGA7 |  |
| COL2A1 | FGF9 | MMRN2 | TIMP1 | ITGA8 |  |
| COL4A1 | FMOD | NELL1 | TIMP2 | ITGA9 |  |
| COL4A2 | FN1 | NELL2 | TIMP3 | ITGA10 |  |
| COL4A3 | FRAS1 | NID1 | TINAG | ITGAM |  |
| COL4A4 | FREM1 | NID2 | TINAGL1 | ITGAV |  |
| COL4A5 | FREM2 | NPNT | TLL1 | ITGAX |  |
| COL4A6 | FREM3 | NTN1 | TNC | ITGB1 |  |
| COL5A1 | HAPLN1 | NTN4 | USH2A | ITGB2 |  |
| COL6A1 | HMCN1 | OGN | VCAN | ITGB3 |  |
| COL6A2 | HMCN2 | OPTC | VTN | ITGB4 |  |
| COL6A3 | HSPG2 | P3H1 | VWA1 | ITGB5 |  |
| COL7A1 | ISLR | P3H2 | VWA2 | ITGB6 |  |
