# Supplementary material for: Basement membrane-related MMP14 predicts poor prognosis and response to immunotherapy in bladder cancer
Source: BMC Cancer. 2024 Jun 19;24:746. doi: 10.1186/s12885-024-12489-y (PMC11186261; doi:10.1186/s12885-024-12489-y)
Supplement: Supplementary file 2 — Supplementary Material 2 [file 12885_2024_12489_MOESM2_ESM.docx]

**Supplementary Table 2. 76 differentially expressed BMRGs between BLCA and normal samples.**

| **gene** | **conMean** | **treatMean** | **logFC** | **pValue** | **fdr** |
| --- | --- | --- | --- | --- | --- |
| COL2A1 | 0.024858 | 2.72224 | 6.774946 | 6.13E-05 | 0.000174 |
| MEP1A | 0.059737 | 1.196383 | 4.323915 | 0.035071 | 0.048869 |
| EVA1A | 0.496632 | 7.106236 | 3.838838 | 4.22E-07 | 2.17E-06 |
| ADAMTS20 | 0.056263 | 0.537074 | 3.254859 | 0.000429 | 0.000936 |
| FBN2 | 0.997726 | 9.343413 | 3.227234 | 0.001015 | 0.002031 |
| GPC2 | 0.670363 | 5.658429 | 3.077387 | 1.47E-07 | 8.05E-07 |
| MMP1 | 23.55925 | 175.8649 | 2.900102 | 0.000729 | 0.001511 |
| LAMC2 | 23.06103 | 171.6814 | 2.896205 | 0.00011 | 0.000303 |
| COL7A1 | 12.94042 | 75.51757 | 2.544928 | 6.70E-06 | 2.37E-05 |
| HAPLN1 | 0.519821 | 2.318012 | 2.156801 | 0.006306 | 0.010751 |
| TINAGL1 | 70.51674 | 242.739 | 1.783368 | 4.94E-06 | 1.79E-05 |
| FRAS1 | 1.059232 | 3.60319 | 1.766257 | 0.016655 | 0.025055 |
| AGRN | 44.32066 | 132.5123 | 1.580075 | 6.53E-08 | 4.11E-07 |
| ACAN | 0.580121 | 1.667644 | 1.523385 | 0.009672 | 0.01581 |
| ADAMTS7 | 3.705232 | 9.579104 | 1.370327 | 0.000253 | 0.000581 |
| ITGA3 | 73.11881 | 168.0643 | 1.200699 | 0.012865 | 0.019882 |
| MMP14 | 195.6391 | 429.4275 | 1.13422 | 0.000232 | 0.000551 |
| ITGB6 | 35.76241 | 77.95504 | 1.124198 | 0.00289 | 0.005341 |
| ITGB4 | 139.9766 | 292.3997 | 1.062756 | 0.001705 | 0.003256 |
| P3H1 | 12.51585 | 25.84995 | 1.046406 | 4.98E-05 | 0.000143 |
| NID1 | 102.0636 | 49.5802 | -1.04163 | 0.019845 | 0.029594 |
| COL13A1 | 3.275942 | 1.588071 | -1.04463 | 0.021392 | 0.031622 |
| FBLN1 | 739.4654 | 349.3839 | -1.08167 | 1.27E-05 | 4.07E-05 |
| LAMB2 | 159.0151 | 74.42187 | -1.09537 | 1.91E-06 | 8.10E-06 |
| TIMP2 | 261.462 | 116.5215 | -1.166 | 1.75E-05 | 5.33E-05 |
| ECM1 | 98.27038 | 43.6045 | -1.17228 | 0.003347 | 0.006118 |
| SERPINF1 | 247.0772 | 109.5167 | -1.17381 | 3.32E-06 | 1.28E-05 |
| COL6A2 | 1005.133 | 413.3029 | -1.28211 | 4.24E-06 | 1.57E-05 |
| FREM3 | 0.154379 | 0.062484 | -1.30492 | 0.000792 | 0.001622 |
| ADAMTS15 | 11.50095 | 4.514171 | -1.34922 | 2.69E-06 | 1.06E-05 |
| EFEMP1 | 134.722 | 52.43441 | -1.3614 | 3.70E-06 | 1.40E-05 |
| NTN1 | 14.67386 | 5.262797 | -1.47935 | 5.18E-08 | 3.42E-07 |
| LAMA4 | 50.87441 | 17.69287 | -1.52377 | 0.00135 | 0.002607 |
| OPTC | 0.331932 | 0.11454 | -1.53504 | 3.93E-07 | 2.09E-06 |
| GPC6 | 16.29313 | 5.469683 | -1.57473 | 1.75E-06 | 7.64E-06 |
| ADAMTS16 | 6.177516 | 2.041938 | -1.59709 | 0.02432 | 0.034743 |
| FBN1 | 44.96713 | 14.79707 | -1.60356 | 0.000699 | 0.001468 |
| EVA1C | 29.60585 | 9.66044 | -1.61572 | 2.39E-09 | 2.71E-08 |
| ADAMTS9 | 11.9198 | 3.600733 | -1.727 | 1.09E-05 | 3.58E-05 |
| FREM1 | 4.642211 | 1.378302 | -1.75192 | 9.37E-07 | 4.43E-06 |
| CSPG4 | 59.96 | 17.75733 | -1.75559 | 0.000249 | 0.000581 |
| ADAMTS5 | 6.584942 | 1.895971 | -1.79623 | 8.06E-08 | 4.87E-07 |
| ITGB3 | 11.61735 | 3.330263 | -1.80257 | 1.70E-05 | 5.24E-05 |
| UNC5C | 4.131174 | 1.094829 | -1.91585 | 8.20E-09 | 7.34E-08 |
| RECK | 14.69871 | 3.682809 | -1.99681 | 3.69E-08 | 2.72E-07 |
| HSPG2 | 129.2795 | 32.05978 | -2.01166 | 0.01138 | 0.017912 |
| ITGA1 | 30.98944 | 7.377154 | -2.07064 | 0.001323 | 0.002586 |
| COL4A6 | 62.16566 | 14.65388 | -2.08484 | 2.02E-06 | 8.36E-06 |
| COL4A4 | 6.321284 | 1.470911 | -2.10351 | 1.19E-06 | 5.47E-06 |
| ITGA9 | 16.02911 | 3.44112 | -2.21974 | 1.06E-07 | 5.98E-07 |
| MATN2 | 99.57164 | 20.83078 | -2.25702 | 1.89E-10 | 4.02E-09 |
| FBLN2 | 209.8324 | 43.72911 | -2.26257 | 9.97E-09 | 8.47E-08 |
| SPON1 | 93.04254 | 18.38296 | -2.33952 | 1.90E-09 | 2.30E-08 |
| ITGA5 | 339.6634 | 67.02485 | -2.34134 | 1.01E-05 | 3.37E-05 |
| PODN | 78.64836 | 14.81003 | -2.40884 | 8.31E-08 | 4.87E-07 |
| LAMA2 | 21.93362 | 4.045113 | -2.43889 | 3.87E-09 | 3.87E-08 |
| FBLN5 | 75.32686 | 13.86107 | -2.44213 | 1.26E-09 | 1.95E-08 |
| LAMC3 | 27.39422 | 4.891065 | -2.48565 | 7.59E-10 | 1.29E-08 |
| THBS1 | 629.6226 | 111.3221 | -2.49975 | 1.68E-05 | 5.24E-05 |
| DDR2 | 40.74017 | 6.630101 | -2.61935 | 1.73E-08 | 1.40E-07 |
| DCN | 414.404 | 67.38329 | -2.62058 | 1.54E-09 | 2.06E-08 |
| FGF9 | 3.292679 | 0.530898 | -2.63276 | 8.14E-09 | 7.34E-08 |
| CCDC80 | 135.5438 | 21.66668 | -2.64521 | 5.24E-08 | 3.42E-07 |
| SLIT2 | 14.10993 | 2.208532 | -2.67555 | 1.58E-09 | 2.06E-08 |
| ADAMTS4 | 66.0034 | 9.738305 | -2.7608 | 0.000105 | 0.000294 |
| MUSK | 0.858632 | 0.125483 | -2.77454 | 2.59E-09 | 2.75E-08 |
| ITGA7 | 74.67923 | 10.73548 | -2.79832 | 2.70E-08 | 2.09E-07 |
| COL14A1 | 98.78151 | 13.52397 | -2.86872 | 3.32E-11 | 1.09E-09 |
| SMOC2 | 173.8248 | 22.95925 | -2.92049 | 2.83E-10 | 5.35E-09 |
| ADAMTS8 | 14.88417 | 1.904993 | -2.96592 | 1.07E-10 | 2.60E-09 |
| SPARCL1 | 934.1194 | 111.619 | -3.06502 | 1.64E-11 | 7.81E-10 |
| SLIT3 | 54.39543 | 5.93229 | -3.19682 | 1.84E-11 | 7.81E-10 |
| ADAMTS1 | 268.4117 | 27.55063 | -3.28429 | 3.37E-12 | 2.86E-10 |
| ITGA8 | 37.84385 | 3.532019 | -3.42149 | 2.54E-12 | 2.86E-10 |
| HMCN2 | 7.388774 | 0.629379 | -3.55333 | 3.93E-08 | 2.78E-07 |
| OGN | 71.86421 | 3.297787 | -4.4457 | 3.85E-11 | 1.09E-09 |
