# Supplementary material for: Basement membrane-related MMP14 predicts poor prognosis and response to immunotherapy in bladder cancer
Source: BMC Cancer. 2024 Jun 19;24:746. doi: 10.1186/s12885-024-12489-y (PMC11186261; doi:10.1186/s12885-024-12489-y)
Supplement: Supplementary file 3 — Supplementary Material 3 [file 12885_2024_12489_MOESM3_ESM.docx]

**Supplementary Table 3. 32 differentially expressed BMRGs had significant prognostic value in BLCA.**

| **id** | **HR** | **HR.95L** | **HR.95H** | **pvalue** |
| --- | --- | --- | --- | --- |
| ADAMTS1 | 1.105563 | 1.00954 | 1.21072 | 0.030404 |
| ADAMTS4 | 1.163561 | 1.059495 | 1.27785 | 0.00153 |
| ADAMTS9 | 1.221082 | 1.057522 | 1.409938 | 0.006485 |
| CCDC80 | 1.182084 | 1.089297 | 1.282776 | 6.05E-05 |
| COL6A2 | 1.121326 | 1.044652 | 1.203629 | 0.001531 |
| COL7A1 | 1.095171 | 1.015417 | 1.18119 | 0.018446 |
| COL14A1 | 1.176727 | 1.075589 | 1.287376 | 0.000386 |
| DCN | 1.102504 | 1.029394 | 1.180807 | 0.005312 |
| ECM1 | 1.19604 | 1.095059 | 1.306331 | 6.96E-05 |
| EFEMP1 | 1.145445 | 1.076233 | 1.219108 | 1.95E-05 |
| FBN1 | 1.305174 | 1.179595 | 1.444123 | 2.47E-07 |
| FBN2 | 1.135846 | 1.059151 | 1.218095 | 0.000355 |
| HSPG2 | 1.220787 | 1.071063 | 1.391441 | 0.002805 |
| LAMA2 | 1.320173 | 1.158886 | 1.503908 | 2.94E-05 |
| LAMA4 | 1.146649 | 1.034586 | 1.270851 | 0.009108 |
| LAMB2 | 1.184281 | 1.046806 | 1.33981 | 0.007219 |
| LAMC3 | 1.135 | 1.002472 | 1.285048 | 0.045615 |
| NID1 | 1.214256 | 1.091601 | 1.350693 | 0.000353 |
| OGN | 1.119924 | 1.01726 | 1.232948 | 0.020954 |
| SERPINF1 | 1.131652 | 1.04089 | 1.230329 | 0.003738 |
| SLIT2 | 1.203164 | 1.074432 | 1.347321 | 0.001358 |
| SMOC2 | 1.105865 | 1.029988 | 1.187331 | 0.005525 |
| SPARCL1 | 1.111511 | 1.024127 | 1.206352 | 0.011385 |
| SPON1 | 1.092922 | 1.016581 | 1.174995 | 0.016168 |
| THBS1 | 1.127793 | 1.03535 | 1.228489 | 0.00585 |
| TIMP2 | 1.146434 | 1.058519 | 1.241651 | 0.000788 |
| CSPG4 | 1.184723 | 1.083777 | 1.295072 | 0.000191 |
| COL13A1 | 1.36705 | 1.126101 | 1.659556 | 0.001575 |
| GPC6 | 1.106639 | 1.010242 | 1.212233 | 0.029323 |
| ITGA5 | 1.163843 | 1.073445 | 1.261854 | 0.000235 |
| MMP14 | 1.186314 | 1.043249 | 1.348997 | 0.009168 |
| UNC5C | 1.400937 | 1.14536 | 1.713543 | 0.001036 |
