# Supplementary material for: Basement membrane-related MMP14 predicts poor prognosis and response to immunotherapy in bladder cancer
Source: BMC Cancer. 2024 Jun 19;24:746. doi: 10.1186/s12885-024-12489-y (PMC11186261; doi:10.1186/s12885-024-12489-y)
Supplement: Supplementary file 4 — Supplementary Material 4 [file 12885_2024_12489_MOESM4_ESM.docx]

**Supplementary Table 4. Top 32 in network string_interactions_short.tsv ranked by MCC method.**

| **Rank** | **Name** | **Score** |
| --- | --- | --- |
| 1 | HSPG2 | 2128 |
| 2 | FBN1 | 2089 |
| 3 | LAMA2 | 1776 |
| 4 | COL6A2 | 1686 |
| 5 | THBS1 | 1662 |
| 6 | DCN | 1553 |
| 7 | ITGA5 | 1512 |
| 7 | NID1 | 1512 |
| 9 | MMP14 | 1398 |
| 10 | LAMA4 | 1128 |
| 11 | ADAMTS1 | 912 |
| 12 | TIMP2 | 792 |
| 13 | ADAMTS4 | 772 |
| 14 | LAMB2 | 722 |
| 15 | LAMC3 | 720 |
| 16 | COL14A1 | 244 |
| 17 | COL7A1 | 196 |
| 18 | FBN2 | 48 |
| 18 | COL13A1 | 48 |
| 20 | ADAMTS9 | 24 |
| 20 | SPON1 | 24 |
| 22 | CSPG4 | 8 |
| 22 | GPC6 | 8 |
| 24 | ECM1 | 7 |
| 25 | SPARCL1 | 5 |
| 25 | OGN | 5 |
| 27 | SERPINF1 | 2 |
| 27 | EFEMP1 | 2 |
| 27 | CCDC80 | 2 |
| 30 | SLIT2 | 1 |
| 30 | UNC5C | 1 |
| 30 | SMOC2 | 1 |
